# Differences in Nutritional and Psychological Habits in Hypertension Patients

**DOI:** 10.1155/2022/1920996

**Published:** 2022-07-08

**Authors:** María Carreira-Míguez, Domingo Jesús Ramos-Campo, Vicente Javier Clemente-Suárez

**Affiliations:** ^1^Faculty of Sport Sciences, Universidad Europea de Madrid, Villaviciosa de Odón 28670 Madrid, Spain; ^2^Departamento de Salud y Rendimiento Humano, Universidad Politécnica de Madrid, Madrid, Spain; ^3^Grupo de Investigación en Cultura, Educación y Sociedad, Universidad de la Costa, Barranquilla, Colombia

## Abstract

Multifactorial factors such as psychological and nutritional habits are controlling factors in hypertension. The aim of the present study was to analyze differences in nutritional and psychological habits in humans with hypertension. Fifty participants with hypertension (HG) (57.5 ± 13.6 years) and 100 participants as control group (CG) with no hypertension disease (48.9 ± 7.9 years) were interviewed via online questionnaire. Multifactorial items in nutrition habits and psychological profile were analyzed by a compendium of questionnaires; psychological measures refer to personality, anxiety, depression, loneliness, perceived stress, and psychological inflexibility; and a nutritional questionnaire to analyze eating habits and nutrition behaviors of the participants. CG showed significantly higher week vitality (*p* = 0.001), juice weekly consumption (*p* = 0.011), coffee weekly consumption (*p* = 0.050), fermented milk weekly consumption (*p* = 0.004), and fruit weekly consumption (*p* = 0.022) than HG. Lower values of weekly coffee consumption and week vitality were found in HG. According to the psychological profile, significant differences were found only depression values, finding HG more depressed than CG (*p* = 0.002). In conclusion, our results showed that people with better nutrition and mental health would present lower levels of blood pressure. Therefore, the combination of psychological therapy and nutritional recommendations for reducing the risk of having hypertension and improving the blood pressure levels may be needed for patient with hypertension.

## 1. Introduction

This article summarizes the current situation of one of the most important 21st century illness, arterial hypertension [1]. It is a problem of increasing prevalence that can be considered an epidemic at present, one of the most common health problems in the world, affects more than 46% of adults, frequently, poorly controlled [2]. Studies carried out in different Spanish populations estimate a prevalence of hypertension that varies between 30 and 50%, but the real percentage is well above that recorded by the Spanish health system, given that it is an underdiagnosed problem, and frequently, poorly controlled [3, 4]. In industrialized societies, like USA, more than 75% aged ≥70 years have hypertension [5]. The American Heart Association estimates that 1.39 billion people worldwide had hypertension in 2010 [6]. In studies carried out in USA in 2012, a high prevalence of hypertension was found (approximately 71 million of people), 48.2% had already been diagnosed, and 36.2% were unaware of the problem (approximately 13 million of people) [7]. Several factors related to nutrition and psychological health can play an important role in regulation of blood pressure. It is known that promoting healthy habits in nutrition and psychological health seems to be quite noteworthy to improve blood pressure. Nutritional habits may be considered as a useful tool to prevent immediate and long-term risk of hypertension in population. Making changes in diet is a good way to help to control high blood pressure [8]. It is very important to create a healthy meal plan because these changes can also help to lose weight and lower heart disease and stroke risk [9]. One of the most important diet recommendations is DASH diet (Dietary Approaches to Stop Hypertension) [10]: this diet is proven to help to lower blood pressure, showing effects sometimes in a few weeks. The main points of DASH diet are limit sodium intake (salt); reduce saturated fat; select monounsaturated oils, such as olive oil, eat foods that are high in soluble fiber; choose fresh fruits and vegetables (5 per day); and eat nuts, seeds, or legumes daily. Choose modest amounts of protein in fish, skinless poultry, and soya products. Other important recommendation is to learn how to shop, cook, and learn to read food labels to choose healthy food. Also, stay away from fast food restaurants, where healthy choices are very difficult to find [11]. In hypertension, psychological factors such as anxiety and depression play a significant role both in its origin and in its evolution [12]. A third of hypertensive patients have major depression [13]. In this line, depressed patients had more cardiovascular diseases and hypertension than other psychiatric patients [14]. Prospective studies have also suggested that depression may be a risk factor for the development of high blood pressure [15]. According to Meng et al., meta-analysis supports that depression is probably an independent risk factor of hypertension, highlighting the necessity to take depression into consideration during the process of prevention and treatment of hypertension [16]. Collazos-Perdomo et al. included in their retrospective cohort study a total of 1721 people, and depression was found as a risk factor for high blood pressure, with a 2-way risk relationship between depression and high blood pressure [17]. Hypertension is a disease with a multifactorial origin, showing patients behavior a direct influence on the symptomatology: but the influence of psychological and nutritional habits has been poorly studied previously. For this reason, we conducted the present research to analyze the differences in nutritional and psychological habits in hypertension patients. We hypothesized that hypertension patients would present different nutrition and psychological profile than healthy control participants.

## 2. Materials and Methods

### 2.1. Participants

In the current study, 50 participants with hypertension (57.5 ± 13.6 years) and 100 participants as control group with no hypertension disease (48.9 ± 7.9 years) were interviewed via online questionnaire. The inclusion criteria for the hypertension group were that participant was diagnosed by hypertension. Hypertension was defined by current use of antihypertensive medications or systolic blood pressure ≥ 140 mm Hg or diastolic blood pressure ≥ 90 mm Hg.

This research complied with the Helsinki declarations (revised in Brazil, 2013) on human research and was approved by the University Ethics Committee (CIPI/18/074). All participants signed an informed consent before the start of the research.

### 2.2. Design and Procedure

Multifactorial items in nutrition habits and psychological profile were analyzed by a compendium of questionnaires, as follows.

#### 2.2.1. Psychological Measures Were Performed by the Following Questionnaires

A short version of the Spanish version of the Big Five Inventory [18], this scale analyses five factors of personality: neuroticism, extraversion, openness, kindness, and responsibility. The reduced version is composed of 10 items that are answered on a 5-point Likert scale, where 1 means completely disagree and 5 means completely agree. An example item is “I see myself as a person who gets nervous easily.” A short version of the Spanish version of Spielberger State-Trait Anxiety Inventory (STAI) [19], composed of 6 items assessing anxiety that are answered on a 4-point Likert scale where 1 means not at all and 4 means very much, was used to measure anxiety. The Acceptance and Action Questionnaire AAQ-II [20] test analyses the psychological inflexibility or experiential avoidance through 7 items, each answered by a 7-point Likert scale, where 0 means never true and 7 means always true. An example item is “Emotions cause problems in my life.” High scores suggest that it is probable that there is current clinical distress. The UCLA Loneliness Scale [21] assesses the measurement of loneliness. In the present study, we used a condensed version composed by three items, each answered by a 3-point Likert scale, where 1 is never and 3 is frequently. An example item is “My interests and ideas are not shared by those around me.” A short Spanish version of Perceives Stress Scale (PSS) [22], this scale assesses the level of perceived stress in a one-month period. It is composed of 14 items answered in a 5-point Likert scale, meaning 0 = never and 4 = very often. An example item is “In the last month, how often did you feel that you could not control important thinks in your life?” The Spanish version of Zung Depression Scale [23] was used to measure depression. The Zung Depression Scale uses a self-applied scale for depression, which has a sensitivity and specificity greater than 80% and consists of 20 items formulated in positive and negative terms. Somatic and cognitive symptoms are highly relevant, with 8 items for each group. The scale also includes 2 items referring to mood and 2 to psychomotor symptoms. It is composed of 4 items, from short time to most of the time.

#### 2.2.2. Nutrition Habits Measures

We used an adapted previously used questionnaire [24] to analyze eating habits and nutrition behaviors in the last 12 months in the population related to weekly consumption frequency of different food groups, including

1. Fruit juices and nectars (250 ml)
2. Alcohol (whiskey, rum, gin…) (50 ml approx.)
3. Beer (250 ml approx.)
4. Wine (50 ml approx.)
5. Soft drinks (coke, soda…) (250 ml approx.)
6. Energy drinks (250 ml approx.)
7. Coffee (250 ml approx.)
8. Tea (250 ml approx.)
9. Milk (250 ml approx.)
10. Fermented products (125 g)
11. Pastries (1 portion)
12. Cookies-sweet cereals(30 g-250 ml)
13. Cheese (50 g)
14. Eggs (1 piece)
15. Meat (150 g)
16. Fish (150 g)
17. Sausage/cold meat (150 g)
18. Legumes (200 g)
19. Rice (150 g)
20. Pasta (150 g)
21. Fruit (1 portion)
22. Raw vegetables (salad) (200 g approx.)
23. Cooked vegetables (200 g approx.)
24. Bread (50 g approx.)
25. Whole grain cereal (bread, rice, oat…) (30 g)
26. Fast food (1 serving)
27. Protein shakes (300 ml)
28. Vitamin supplements (1 capsule)

Each item was ranged from 1 to 6, where 1 means “I do not consume,” 2 means “less than three times per week,” 3 means “three times or more per week,” 4 means “seven or more times per week,” 5 means “ten or more times per week,” and 6 means “more than thirteen times per week.”

We also evaluated the vitality during the week and at the end of the week in two questions each answered by a 10-point Likert scale, where 0 means very low and 10 means very high. For the question “Do you have Migraine headache?” is answered by a 10-point Likert scale, where 0 means rarely got it and 10 means very often. For the question “How satisfied do you feel with your weight?,” the answers were “completely satisfied,” “I would like to increase weight,” and “I would like decrease weight.” For the question “How many glasses of water do you drink per day (250ml)?,” the answers ranged from “0” to “more than 10.” For the question “How many sugar spoons takes per day?,” the answers ranged from “0” to “more than 5.” For the question “How do you have post-meal digestion?,” the answers were “I feel good normally,” “sometimes I feel hard, heavy digestion,” and “very often I feel hard, heavy digestion.” For the question “In the last week, which type of feces did you have?” were 1 means “shard chunks,” 2 means “sausage shape,” 3 means “noodle form,” 4 means “snakes,” 5 means “soft chunks,” 6 means “soft chunks with undone limits,” and 7 means “watery with no solid chunks” we used Bristol Scale [25].

### 2.3. Statistical Analysis

The statistical analysis was carried out using the Statistical Package for the Social Sciences (SPSS) version 24.0 (SPSS Inc., Chicago, IL, USA). Descriptive statistics (mean and standard deviation) were calculated for each variable. To analyze group differences between control group and hypertension group, an independent *T* test was conducted. The significance level was *p* ≤ 0.05.

## 3. Results and Discussion

### 3.1. Results

Table 1 shows the results of the nutritional variables. CG showed significantly higher week vitality (*p* = 0.001), juice weekly consumption (250 ml) (*p* = 0.011), coffee weekly consumption (250 ml) (*p* = 0.050), fermented milk weekly consumption (125 g-ml) (*p* = 0.004), and fruit weekly consumption (1 unit) (*p* = 0.022) HG. Lower values of coffee weekly consumption and week vitality were found in HG.

According to the psychological profile, significant differences were found only in the Zung Depression Scale variable, showing HG higher values than CG (*p* = 0.001) (Table 2).

## 4. Discussion

The aim of the present study was to analyze differences in nutritional and psychological profiles of hypertensive patients and a control healthy participants. The initial hypothesis was realized when behavioral differences between hypertense and control groups were observed.

Previous evidence showed that multifactorial factors such as psychological and nutritional habits are controlling factors in development disease. Therefore, as a result, we hypothesized that people with better nutrition and mental health would present lower levels of blood pressure, and the initial hypothesis was confirmed.

According to the dietary habits, some differences were found between both groups. CG presented significantly higher values in week vitality, juice, coffee, fermented milk, and fruits weekly consumption, than HG. Nonsignificant differences were found in the rest of nutritional variables analyzed. In this line, overweight and blood hypertension presented a direct relationship, showing the importance of nutritional habits and physical activity, highlighting that these factors that occur at an earlier age [26]. To support governments in strengthening the prevention and control of cardiovascular disease, WHO and the United States Centres for Disease Control and Prevention provide a strategic approach to improve cardiovascular health in countries across the world, limiting the intake of foods high in saturated fats, eating more fruit and vegetables, and eliminating/reducing trans fats in diet [2]. In Spain, government promoted the campaign “5 a day.” It is a worldwide movement endorsed by the World Health Organization (WHO) that promotes the consumption of a minimum of 5 servings of fruits and vegetables a day as the basis of a healthy diet. The campaign slogan refers to the importance of eating a minimum of five daily servings of fruit and vegetables for the correct nutrition of children. In addition, they served to prevent diseases that appear in adulthood, such as hypertension and cardiovascular problems [27].

The lower values of week vitality in the HG could be related with the higher use of medication in this group, since previous authors suggested that the effects of drug treatment were most marked in patients who had had previous antihypertensive medications. Antihypertensive medication has a direct impact in quality of life, well-being, and vitality of patients [28, 29]. Recommendations of low caffeine ingestion, especially coffee, were found full filet by the HG, with lower values of caffeine consumption. This was in line with previous studies where the risk of hypertension was lower in coffee abstainers, showing how coffee ingestion around 5 cups per day causes a small elevation in blood pressure when compared to abstinence or use of decaffeinated coffee [30]. However, other studies' findings on coffee consumption and its association with the incidence of hypertension are not homogeneous and still inconsistent [31]. In this line, juice, fermented milk, and fruit weekly consumption were higher in CG than in HG. Previous research stated that modifications in lifestyle must be recommended to hypertensive patients, with strategies limiting salt and alcohol, fruits, vegetables, and low-fat milk products to obtain a suitable weight [32].

In the present study, HG showed higher depression levels than CG. This result was in line with previous research that reported the prevalence of depression in hypertensive patients [13]. In this line, the inclusion of nutritional recommendations in hypertension achieved physiological modifications that allow a reduction of hypertension [33]. Modifications in nutritional habits played an important role, since they present a large anti-inflammatory effect on the organism, being able to prevent depression disorders. Thus, diet is a common source of inflammation, and inflammation is associated with depression. In 8 years, study that included 3648 participants (1577 males and 2071 females) was found how proinflammatory diet may be associated with higher incidence of depressive symptoms, recommending an anti-inflammatory diet since it could reduce depression risk [34]. Cross-sectional studies indicated that individuals with depression consumed more proinflammatory foods and lower anti-inflammatory nutrients than the general population [35]. In the same line, poor diet produces an increased activation of the sympathetic autonomic nervous system [8], being the autonomous nervous system considered as an important factor in the genesis and development of arterial systemic hypertension [36]. The mechanisms and causes of the increased sympathetic activity are not very well known, but behavioral and other lifestyle factors like nutritional factors and health care appear to be involved [37]. Changing nutritional habits produced an increase of parasympathetic system activity and, in fact, a decrease of blood pressure levels. Depression is common in patients with uncontrolled hypertension and may interfere with blood pressure control. Screening for depression in hypertensive patients is a simple and cost-effective tool that may improve outcomes [38]. Because hypertension and depression share common pathways, it is possible that each disease has an impact on the natural history of the other. Greater healthcare utilization among patient depression may contribute to faster hypertension control [39].

## 5. Conclusions

We found how hypertense patients presented lower consumption of fruits, juices, and fermented milk products, lower vitality, and higher levels of depression. The results of this study open a new multidisciplinary field in the treatment of hypertension, so present in the world today. The combination of psychological therapy and nutritional recommendations could be and efficient tool to decrease the risk of hypertension in futures generations and to improve blood pressure in patient with diagnosed hypertension. Finally, future prospective observational studies are needed to establish the causal role of diet factors more clearly in the development and progression of hypertension.

## Figures and Tables

**Table 1:** Nutritional variables.

Variable	Hypertensive group	Control	*T*	*p*	Confidence	Interval
					Lower	Upper
Water glasses per day (250 ml)	2.6 ± 1.1	2.7 ± 1.4	0.136	0.892	-0.41	0.47
Week vitality (0-10)	6.9 ± 1.8	7.7 ± 1.4	3.328	0.001	0.36	1.42
Vitality at the end of week (0-10)	6.1 ± 2.5	6.6 ± 2.4	1.291	0.199	-0.29	1.37
Migraine headache occurrence (0-10)	3.1 ± 3.4	2.1 ± 2.9	-1.797	0.074	-2.01	0.09
Juice weekly consumption (250 ml)	1.6 ± 0.8	2.1 ± 1.2	2.571	0.011	0.11	0.85
Distilled alcohol weekly consumption (50 ml)	1.3 ± 0.6	1.3 ± 0.5	0.426	0.670	-0.14	0.22
Bear weekly consumption (250 ml)	1.9 ± 1.1	1.9 ± 1.0	0.203	0.840	-0.33	0.40
Wine weekly consumption (50 ml)	1.7 ± 1.0	1.7 ± 0.7	0.410	0.683	-0.24	0.36
Soft drink weekly consumption(250 ml)	1.5 ± 0.9	1.4 ± 0.8	-0.829	0.408	-0.41	0.17
Energy drink weekly consumption (250 ml)	1.10.3±	1.1 ± 0.4	0.227	0.821	-0.11	0.14
Coffee weekly consumption (250 ml)	1.8 ± 1.2	2.7 ± 1.5	1.979	0.050	0.00	1.06
Tea weekly consumption (250 ml)	1.6 ± 0.8	1.8 ± 1.2	1.285	0.201	-0.13	0.63
Milk weekly consumption (250 ml)	2.5 ± 1.3	2.4 ± 1.4	-0.302	0.763	-0.55	0.40
Fermented milk product weekly consumption (125 g-ml)	2.1 ± 1.1	2.7 ± 1.3	2.969	0.004	0.22	1.12
Pastry weekly consumption (1 portion)	1.7 ± 0.9	1.8 ± 1.0	0.503	0.616	-0.27	0.45
Cookie-sweet cereals weekly consumption (30 g-ml)	1.8 ± 1.1	2.0 ± 1.2	0.917	0.361	-0.22	0.61
Cheese weekly consumption (50 g)	2.3 ± 1.0	2.5 ± 1.1	0.751	0.454	-0.23	0.51
Egg weekly consumption (1 piece)	2.4 ± 0.7	2.6 ± 0.8	1.446	0.150	-0.07	0.47
Meat weekly consumption (150 g)	2.4 ± 0.8	2.7 ± 1.0	1.671	0.097	-0.05	0.59
Fish weekly consumption (150 g)	2.5 ± 0.8	2.5 ± 0.8	-0.217	0.829	-0.32	0.25
Sausage weekly consumption (50 g)	1.9 ± 1.0	2.1 ± 1.0	1.059	0.291	-0.16	0.54
Legume weekly consumption (200 g)	2.4 ± 0.8	2.4 ± 0.8	-0.166	0.868	-0.30	0.25
Rice weekly consumption (150 g)	2.0 ± 0.6	2.2 ± 0.7	1.220	0.225	-0.09	0.37
Pasta weekly consumption (150 g)	2.0 ± 0.5	2.1 ± 0.7	1.422	0.157	-0.06	0.37
Fruit weekly consumption (1 portion)	3.0 ± 1.2	3.6 ± 1.5	2.317	0.022	0.08	1.06
Fresh vegetable-salad weekly consumption (200 g)	3.0 ± 1.2	3.2 ± 1.3	1.253	0.212	-0.17	0.74
Cooked weekly consumption (200 g)	3.0 ± 1.2	3.1 ± 1.3	0.791	0.430	-0.27	0.62
Bread weekly consumption (50 g)	2.7 ± 1.2	3.1 ± 1.4	1.545	0.125	-0.10	0.84
Whole grain-rice-oat weekly consumption (30 g)	2.1 ± 1.1	2.1 ± 1.1	-0.225	0.822	-0.44	0.35
Fast food-pizza-hamburger weekly consumption (1 serving)	1.5 ± 0.6	1.6 ± 0.7	1.238	0,218	-0.09	0.38
Protein shake weekly consumption (300 ml)	1.2 ± 0.5	1.1 ± 0.4	-1.185	0.238	-0.08	0.06
Vitaminic supplement weekly consumption (1 capsule)	1.4 ± 10	1.4 ± 0.9	-0.498	0.619	-0.40	0.24
Daily teaspoons of sugar	1.4 ± 0.5	1.4 ± 0.6	-0.131	0.896	-0.22	0.20
Postmeal digestion (1-5)	1.4 ± 0.6	1.3 ± 0.5	-1.072	0.285	-0.30	0.09
Bristol scale	3.7 ± 1.1	3.7 ± 1.1	0.074	0.941	-0.37	0.40

**Table 2:** Results of psychological variables analyzed.

Variable	Hypertensive group	Control	*T*	*p*	Confidence	Interval
					Lower	Upper
Zung	49.0 ± 6.2	45.6 ± 5.1	-3.513	0.001	-5.20	-1.46
PSS4	13.2 ± 3.6	12.8 ± 4.0	-0.626	0.532	-1.75	0.91
STAI	13.2 ± 3.6	12.8 ± 4.0	-0.626	0.532	-1.75	0.91
UCLA	4.5 ± 1.5	4.1 ± 1.3	-1.866	0.064	-0.93	0.03
AAQII	22.3 ± 10.5	19.5 ± 9.1	-1.649	0.101	-6.02	0.54
B5_extraversion	5.6 ± 2.0	5.8 ± 1.7	0.563	0.575	-0.45	0.81
B5_agreeableness	7.0 ± 1.6	6.5 ± 1.4	-0.954	0.342	-0.77	0.27
B5_conscientiousness	7.4 ± 1.6	7.8 ± 1.6	1.717	0.088	-0.07	0.97
B5_neuroticims	6.2 ± 2.0	6.0 ± 1.9	-0.561	0.576	-0.86	0.48
B5_open to experience	7.7 ± 1.9	7.2 ± 1.8	-1.752	0.082	-1-17	0.70

Zung Depression Scale variable; PSS4: Perceives Stress Scale; STAI: Spielberger State-Trait Anxiety Inventory; UCLA: UCLA Loneliness Scale; AAQII: Acceptance and Action Questionnaire II; B5: Big Five Factors (extraversion, agreeableness, conscientiousness, neuroticism, and openness to experience).

## Data Availability

All data are present in the manuscript.
